# Monitoring the macrophage response towards biomaterial implants using label-free imaging

**DOI:** 10.1016/j.mtbio.2023.100696

**Published:** 2023-06-13

**Authors:** Chuan-en Lu, Ruth E. Levey, Giulio Ghersi, Nathan Schueller, Simone Liebscher, Shannon L. Layland, Katja Schenke-Layland, Garry P. Duffy, Julia Marzi

**Affiliations:** a Institute of Biomedical Engineering, Department for Medical Technologies and Regenerative Medicine, Eberhard Karls University Tübingen, Tübingen, Germany; b Anatomy and Regenerative Medicine Institute (REMEDI), School of Medicine, University of Galway, Ireland; c ABIEL Srl, C/o ARCA Incubatore di Imprese, Palermo, Italy; d Department of Biological, Chemical and Pharmaceutical Sciences and Technologies, University of Palermo, Italy; e NMI Natural and Medical Sciences Institute at the University of Tübingen, Reutlingen, Germany; f Cluster of Excellence IFIT (EXC 2180) “Image-Guided and Functionally Instructed Tumor Therapies”, Eberhard Karls University Tübingen, Tübingen, Germany; g Science Foundation Ireland Centre for Research in Medical Devices (CÚRAM), University of Galway, Ireland

**Keywords:** Foreign body response, Raman imaging, Extracellular matrix, Fibrosis, Diabetes

## Abstract

Understanding the immune system's foreign body response (FBR) is essential when developing and validating a biomaterial. Macrophage activation and proliferation are critical events in FBR that can determine the material's biocompatibility and fate in vivo. In this study, two different macro-encapsulation pouches intended for pancreatic islet transplantation were implanted into streptozotocin-induced diabetes rat models for 15 days. Post-explantation, the fibrotic capsules were analyzed by standard immunohistochemistry as well as non-invasive Raman microspectroscopy to determine the degree of FBR induced by both materials. The potential of Raman microspectroscopy to discern different processes of FBR was investigated and it was shown that Raman microspectroscopy is capable of targeting ECM components of the fibrotic capsule as well as pro and anti-inflammatory macrophage activation states, in a molecular-sensitive and marker-independent manner. In combination with multivariate analysis, spectral shifts reflecting conformational differences in Col I were identified and allowed to discriminate fibrotic and native interstitial connective tissue fibers. Moreover, spectral signatures retrieved from nuclei demonstrated changes in methylation states of nucleic acids in M1 and M2 phenotypes, relevant as indicator for fibrosis progression. This study could successfully implement Raman microspectroscopy as complementary tool to study in vivo immune-compatibility providing insightful information of FBR of biomaterials and medical devices, post-implantation.

## Highlights

• ECM proteins in the fibrotic capsule were visualized marker-independently.
• Spectral signatures discriminate fibrotic collagens and connective tissue.
• Raman imaging enables macrophage phenotyping ex vivo.

## Graphical abstract

**Figure:**
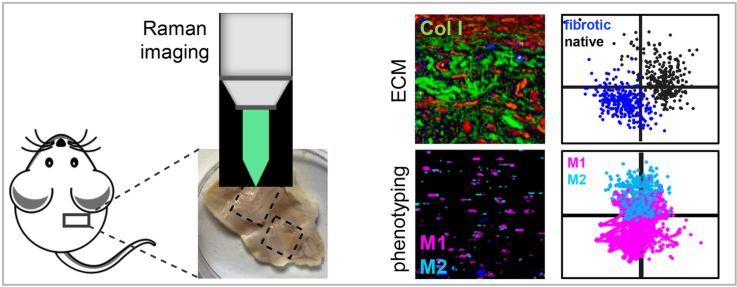

## 1 Introduction

Type 1 diabetes (T1D) is an autoimmune disease that induces the destruction of pancreatic islets, resulting in the deficiency of insulin secretion [1]. Current treatments still mainly rely on daily extraneous insulin regimens, which can negatively affect the quality of life for patients suffering from T1D and is difficult to manage proper glycemic control over an entire lifespan [[2], [3], [4], [5]]. Alternative strategies such as continuous subcutaneous insulin infusion (CSII) therapy [6,7], insulin pumps in combination with real-time glucose monitoring systems [8] or partially closed-looped insulin delivery systems [9] have been developed and are in clinical use. Pancreatic islet transplantation provides a potential method to cure T1D; however, the positive therapeutic outcomes of insulin independence typically only lasts 5 years. The foreign body response (FBR) driven by immune cell infiltration can influence the ultimate therapeutic outcome of the implantable devices, including islet encapsulation devices [[10], [11], [12], [13]]. FBR is an end stage process of the wound healing and pathogen removal processes, which can cause damage to a material or facilitate the immunological process to engulf an implant into a fibrotic capsule [[14], [15], [16]]. The fibrotic capsule can physically wall off the device and impede it from oxygen, nutrients and insulin transportation, thereby impairing the functionality and survival rate of the engrafted insulin-producing cells.

FBR drives tissue fibrosis. It is initiated by non-specific serum protein adsorption to the implant surface followed by neutrophil and monocyte recruitment [17]. Once monocytes adhere to the interface, they start differentiating into macrophages which play a crucial role in modulation of the inflammatory reaction, ECM remodeling and wound healing [17]. Macrophages can undergo further polarization towards different stages of wound healing processes. Macrophages can differentiate to highly heterogeneous subpopulations among which pro- and anti-inflammatory phenotypes are the most relevant ones: the non-activated macrophages (M0), the classically activated macrophages which are related to proinflammatory reaction (M1) and alternatively activated macrophages which are associated with regenerative and anti-inflammatory cascades (M2) [18]. The balance between M1 and M2 macrophages is essential to tissue remodeling, the imbalance between the phenotypes can result in negative effect of scar tissues formation [19]. Domination of M1 phenotype population has been shown to be detrimental to the implantation [20]; whereas M2 macrophages can secrete factors (e.g. IL-10, RELMα, Arg-1) that support tissue remodeling and inhibit fibrosis [21]. Therefore, the therapeutic strategies for implantable devices aim to reduce the ratio of M1/M2 [20,21]. Over time, macrophages fuse into foreign body giant cells, which induce fibroblasts and myofibroblasts and to migrate to the implantation site where they secrete collagens and other ECM components finally forming a fibrotic capsule surrounding the implant [19].

FBR is typically evaluated using histological staining such as H&E and Masson's trichrome which require invasive procedures and further sectioning protocols. Moreover, most histological stains are only limited to the qualitative observation of tissue and fiber morphologies. Therefore, Raman microspectroscopy (RMS) was employed to investigate FBR at the interface between the tissues and biomaterials. RMS is a non-destructive and marker-independent analytical technique that can identify molecular information based on the interaction between the laser light and the chemical bonds in a sample [22]. Recently, our group published a study using micro-computed tomography (μCT) and RMS to investigate the fibrotic capsule caused by an implantable therapeutic reservoir on a streptozotocin (STZ)-induced diabetic animal model [23]. We demonstrated the capability of RMS to identify the presence of advanced glycation end-products (AGEs) in Collagen I (Col I) in the diabetic animals. In this study, RMS was applied to the resulting capsular areas which were induced and surrounded enveloping two biomaterial candidates for islet encapsulation in vivo, polyvinylidene fluoride (PVDF) and TPU-chronoflex, to further gain a deeper understanding of FBR in a STZ-induced diabetes model. Specifically, RMS was used to discriminate interstitial connective tissues and the fibrotic capsule via Col I spectra in combination with multivariate tools. Numerous studies categorizing tissue types via RMS have been published, especially in the field of tumor biology, cell malignancy and carcinoma identification [24,25]. However, there is little research regarding ECM content differentiation between normal tissues and fibrotic capsules with RMS. Furthermore, RMS was used to detect the differences in methylation states between M1/M2 macrophages. Recent research from our group revealed RMS with multivariate analysis could distinguish different subtypes of macrophages (M0, M1, M2a and M2c) in situ through their lipid spectra [26]. This work was supported by other studies focused on macrophage phenotypes identification via RMS [[26], [27], [28], [29]]. Most of these studies conducted their measurements on in-vitro samples. This study is the first to focus on performing RMS to classify macrophages phenotypes in a more complex, three-dimensional tissue environment following medical device implantation.

## 2 Materials and methods

### 2.1 Biomaterials

Briefly, TPU-chronoflex [23] and PVDF sheets were produced by an in-house electrospinning approach with thickness of 50 ​μm and pore size of 2 ​μm. The TPU-chronoflex pouches were generated with dual layers of TPU and chronoflex sheets. The PVDF pouches were produced with dual layers of PVDF sheets (Supplementary Fig. S1A). Pouches were manufactured by using in-house laser welding techniques developed by Boston Scientific (Galway, Ireland). Empty pouches were used for implantation in this study.

### 2.2 Surgical procedure, implantation and explantation

The procedures of biomaterial implantation have been described previously and were shown in Supplementary Fig. S1B) [23]. Animal studies were conducted by Abiel Sr (Palermo, Italy) with the approval of the Italian Ministry of Health (Authorization No. 66/2017-PR). Six female RccHan Wistar rats (150/200 ​g, 12-week-old, ENVIGO) were utilized for the study. All animals were treated with 65 ​mg/kg STZ intravenously to induce diabetes 14 days prior to implantation. Every rat underwent anesthetization by isoflurane before and during implantation. PVDF and TPU-chronoflex pouches were implanted subcutaneously. Each rat was implanted with two pouches of the same biomaterial type on the mid-back of the rat. Each type of pouch was implanted in 3 animals. On day 15, the biomaterials with surrounding skin tissues were explanted en bloc. 4% paraformaldehyde was used to fix the tissue blocks overnight at 4 ​°C before paraffin embedding. The tissues were sectioned to 5 ​μm thick slices and placed on glass slides. Before histological staining and Raman imaging, sections were deparaffinized in 100% xylene and rehydrated with a descending ethanol row. Skin tissues from regions without implants served as internal controls.

### 2.3 Histological staining

#### 2.3.1 Modified Movat pentachrome staining

Deparaffinized sections were stained with a histologic pentachrome stain. Briefly, Weigert's resorcin-fuchsin solution (Banishes Fuchsin, Waldeck GmbH, Münster, Germany plus ferric chloride, Carl Roth GmbH, Karlsruhe, Germany in 96% acetified ethanol) was applied for 30 ​min and washed by running tap water for 2 ​min. 80% ethanol solution was used for the first color differentiation. Weigert's Iron hematoxylin (Waldeck GmbH) plus ferric chloride was applied for 10 ​min to stain nuclei and washed by deionized water. The second differentiation was conducted by 0.5% HCl–EtOH, followed by running tap water for 10 ​min. The sections were treated with 3% acetic acid (Carl Roth GmbH) first and then stained with 1% alcian blue (Waldeck GmbH) followed by Brilliant crocein acid-fuchsin (Brilliant crocein y plus Acid-fuchsin, both Waldeck GmbH).1% Acetic Acid and 5% phosphorus tungstic acid (Aldrich, USA) were employed for the final differentiation. The sections were then drained with 100% ethanol several times, the collagens were stained with a saffron du gâtinais solution (Waldeck GmbH). Finally, the sections were drained in 100% ethanol several times followed by incubation in Roti-Histol (Carl Roth GmbH) before mounting in Roti-Histokitt (Carl Roth GmbH) The stained sections were imaged by utilizing a light microscope (Observer Z1, Carl Zeiss AG, Oberkochen, Germany). The fibrotic capsule thickness was examined via the measurement function in Zen 2 blue edition (Carl Zeiss AG). The measurement of capsular thickness is depicted in Supplementary Fig. S2.

#### 2.3.2 Picrosirius red staining

Deparaffinized sections were washed with distilled water for 5 ​min. Nuclei were stained with Weigert's hematoxylin (Waldeck GmbH) for 8 ​min. The stained sections were then rinsed with running tap water for 10 ​min. Picrosirus red (Sirius red plus picric acid solution, Sigma Aldrich USA) solution was applied for 1 ​h for collagen staining. Sections were washed with 0.5% acetic acid followed by absolute ethanol. The stained sections were imaged via polarized light microscopy (Axio Observer Z1, Carl Zeiss AG). The resulting colors vary between different thicknesses of collagen fibers, which has been reported to be related to collagen fiber maturity [30,31]. Red to orange colors indicate thicker fibers; yellow to green colors illustrate thin collagen fibers. Hue, saturation, brightness (HSB) color model in the Image J (Fiji version 2.0.0) software was utilized for analysis. Settings were adapted based on parameters previously reported [30,32]. Red and orange (red: 1–13; orange: 14–25) was determined to represent mature collagens whereas yellow and green colors indicated immature collagens (yellow: 26–52; green: 53–110) [30,31]. The area percentages of red/orange to yellow/green were quantified. Three images were acquired and analyzed for each sample.

### 2.4 Immunofluorescence staining

Deparaffinized sections were stained for the following proteins: mouse monoclonal *anti*-αSMA (1:500; Sigma-Aldrich, St. Louis, USA), rabbit polyclonal anti-Col I (1:500; Cedarlane, Burlington, Ca) and rabbit polyclonal anti-Col III (1:75; Acris, Herford, Germany) as described previously [23]. In addition, tissue sections were stained for macrophage surface proteins: mouse monoclonal anti-CD68 (1:50; Bio-Rad, Hercules, CA, Cat#: MCA341R), rabbit monoclonal *anti*-CCR7 (1:250; abcam, Cambridge, United Kingdom, Cat#: ab32527), and rabbit polyclonal anti-CD204 (1:200; ThermoFischer, Waltham, MA, Cat#: PA5-102519). All primary antibodies were incubated overnight at 4 ​°C. AlexaFluor 594 conjugated goat anti-mouse (1:250, Thermo Fisher, Waltham, MA) was applied as secondary antibody for CD68 or αSMA, and AlexaFluor 488 conjugated goat anti-rabbit (1:250; Thermo Fisher, Waltham, MA) for *anti*-CCR7, anti-CD204, anti-Col I or anti-Col III antibodies. Control slides received equivalent volumes of dilution buffer. Fluorescence images were obtained by confocal laser scanning microscopy (LSM 880, Carl Zeiss AG). The cell numbers were counted manually by using the colocalization analysis (Zen 2 Blue, Carl Zeiss AG). Colocalization analysis is conducted on a pixel-by-pixel basis, the threshold of the scattering plot of pixel intensities of each channel was set to cover the frequency of 255. Control tissue sections without primary antibodies were used to validate the IF signals. Triplicate images were selected for each sample. CD68^+^/CCR7^+^ or CD68^+^/CD204^+^ cells were indicated as M1-like or M2-like macrophages, respectively. Mean gray value (MGV) was employed to quantify the expression level of the ECM components by using ImageJ V 1.52p, which is the amount of the gray values of all pixels normalized by the number of pixels.

### 2.5 Raman microspectroscopy and imaging

Raman microspectroscopy (WITec alpha 300 ​R, Ulm, Germany) was performed at the interface of the fibrotic capsule as described previously [23]. A 63× objective (W Plan-Apochromat 63×/1.0 M27, Carl Zeiss AG) was used to image deparaffinized and hydrated tissue sections, sequential to sections subjected to IF staining. Spectral preprocessing and analysis were conducted in Project Five 5.2 (WITec GmbH). True component analysis (TCA) was applied to generate false-color coded intensity distribution heatmaps based on reference spectra of αSMA, Col I, Col III and nuclei generated in a previous study [23]. To obtain reference spectra of immune cells, Raman imaging was correlated with CD68/CCR7 or CD68/CD204 positive immunofluorescence images and spectra from co-localized pixel were extracted. Triplicate images were selected for each sample. Similar to ECM signatures, the retrieved spectra were used as reference spectra to identify macrophage polarization states via TCA. MGV was also performed to quantify the signal intensities of the specific proteins. The cell numbers were counted manually by using ImageJ V 1.52p.

### 2.6 Multivariate data analysis

Biological molecular information was analyzed and interpreted by principal component analysis (PCA) (The Unscrambler X, CAMO Software AS, Oslo, Norway). Col I spectra were extracted from defined regions within the fibrotic capsule and interstitial connective tissue. Spectral data from normal skin tissues were extracted as controls. PCA analysis was used to investigate the molecular fingerprints in Col I spectra based on Raman shifts between different types of tissues. Single spectra information were visualized in 2D scatter plots and averaged per animal for visualization and statistical comparison in box plots. For macrophage identification, nuclei spectra of immunofluorescence stained CCR7 positive (CCR7^+^) cells or CD204 positive (CD204^+^) cells were extracted and applied to non-stained fibrotic capsule Raman scans to assess the difference between M1 and M2 phenotypes. The ratio of M1 to M2 macrophages was calculated manually based on the numbers of nuclei positive for CCR7^+^ or CD204^+^ derived reference spectra. PCA analysis was performed of the two macrophage subsets for further biological interpretation.

### 2.7 Statistics

Statistical analyses were performed using Prism 9 (GraphPad, La Jolla, USA) software. Unpaired t-tests were conducted for comparison between two experimental groups. One-way parametric ANOVA tests were applied to compare fibrotic tissues and interstitial connective tissues. Significance was defined as p ​< ​0.05 and indicated in the figures as ∗p ​< ​0.05, ∗∗p ​< ​0.01, ∗∗∗p ​< ​0.001, and ∗∗∗∗p ​< ​0.0001. N numbers were defined as biological replicates which were 3 animals per implant material and 6 (pooled TPU and PVDF data) for macrophage comparison.

## 3 Results

### 4.1 PVDF and TPU-chronoflex pouches induce different thickness of fibrotic capsule with similar collagen state

**Fig. 1:**
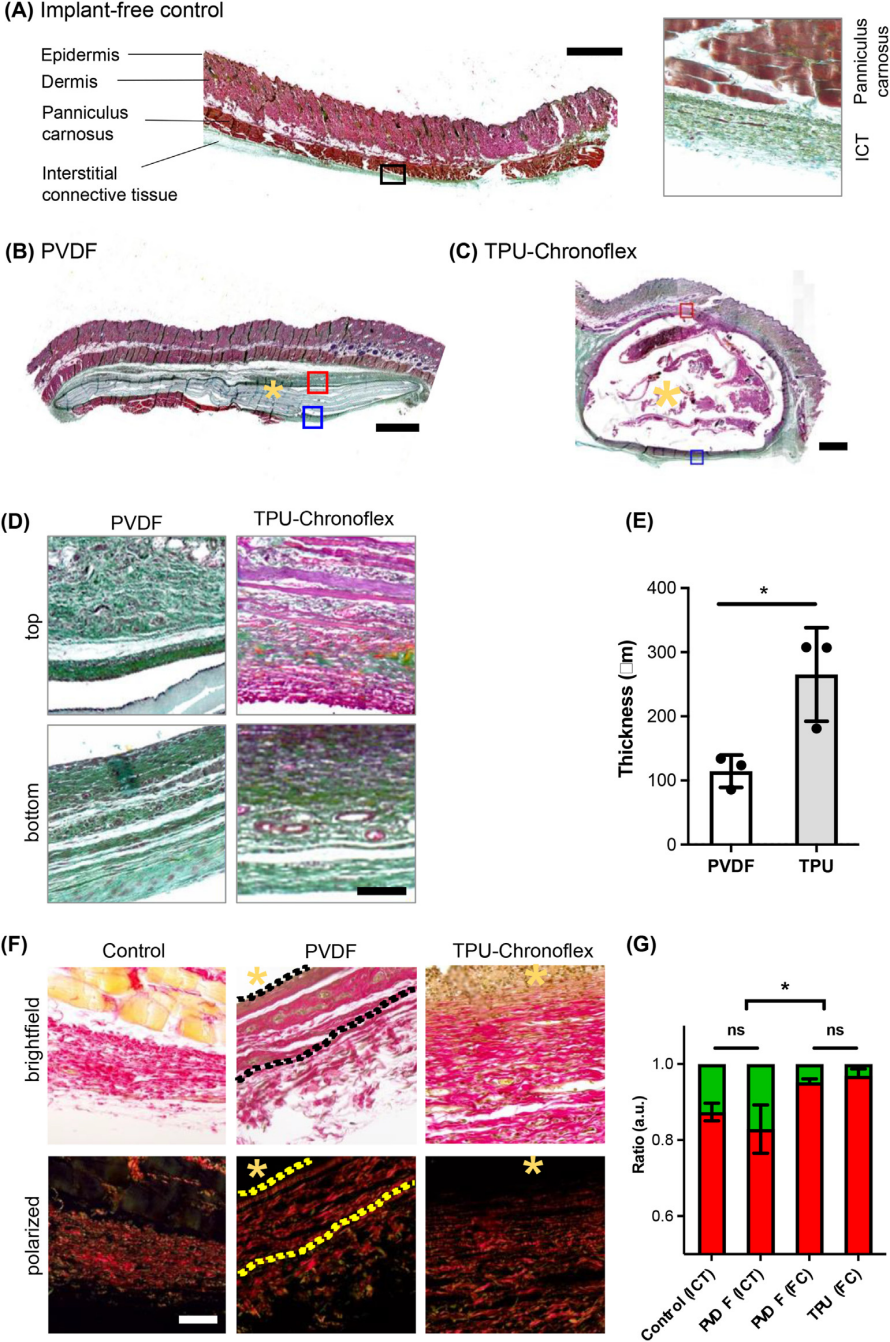
**Modified Movat pentachrome and picrosirius red stains visualize tissue morphology and collagen fibers of fibrotic capsules.** Histological overview images of **(A)** implant-free rat skin tissues and after subcutaneous **(B)** PVDF or **(C)** TPU-chronoflex implantation. Modified Movat pentachrome; light scarlet red: Epidermis and dermis; dark scarlet red: panniculus carnosus; yellow: collagen; blue: proteoglycans. Green: colocalization of collagen (yellow) and proteoglycans (blue); yellow asterisk: implant region; scale bars equal 1 ​mm (left); **(D)** Selected ROIs of top and bottom regions of capsular areas for PVDF and TPU-chronoflex tissues; Scale bar equals 50 ​μm ​**(E)** Average thickness of the fibrotic capsule was determined for both implants. N ​= ​3, *t*-test, p∗ ≤0.05 **(F)** Picrosirius red staining demonstrated red/orange collagen fibers and randomly distributed yellow/green fibers in the fibrotic tissues. Scale bar equals 50 ​μm. **(G)** The ratio of red/orange to yellow/green fibers showed significant difference between interstitial connective tissue (ICT) and fibrotic capsule (FC). Yellow asterisk: implant side. (For interpretation of the references to colour in this figure legend, the reader is referred to the Web version of this article.)

PVDF and TPU-chronoflex pouches were implanted in rats subcutaneously and explanted after 15-days followed by paraformaldehyde fixation, sectioning and paraffin embedding. The overall ECM composition within the skin tissues was visualized via Movat pentachrome staining**.** Epidermis and dermis were illustrated in light scarlet red. Panniculus carnosus (striated muscle) was shown in dark scarlet red; ground substance of interstitial connective tissue and the dense fibrotic capsule around the implant appeared in greenish, which display the colocalization of collagen (yellow) and proteoglycans (blue). Nuclei were stained in dark purple. Implant-free tissue extracted from back regions in the periphery of the transplants showed natural interstitial connective tissues under the panniculus carnosus (Fig. 1A). Subcutaneous tissues with the implants demonstrated a higher degree of morphological changes due to fibrotic capsule formation**.** Visualization of subcutaneous tissues with PVDF implantation (Fig. 1B) showed relatively thinner layers of fibrotic capsule compared to the TPU-chronoflex group (Fig. 1C). Picrosirius red stained sections showed the collagen fiber architecture when exposed to polarized light due to birefringent features of collagens (Fig. 1D). The thickness of the capsule around TPU-chronoflex was 265.3 ​± ​42.06 ​μm compared to 114.5 ​± ​14.62 ​μm in PVDF (Fig. 1E). The quantification of picrosirius colorimetry demonstrated significant difference between fibrotic capsule and interstitial connective tissue (Fig. 1F and G). Within capsule regions, the ratio of red/orange to yellow/green fibers showed no significant difference. Compared to interstitial connective tissue, contribution of red/orange fibers was higher in the capsular areas.

### 4.2 ECM characterization of fibrotic capsule regions

**Fig. 2:**
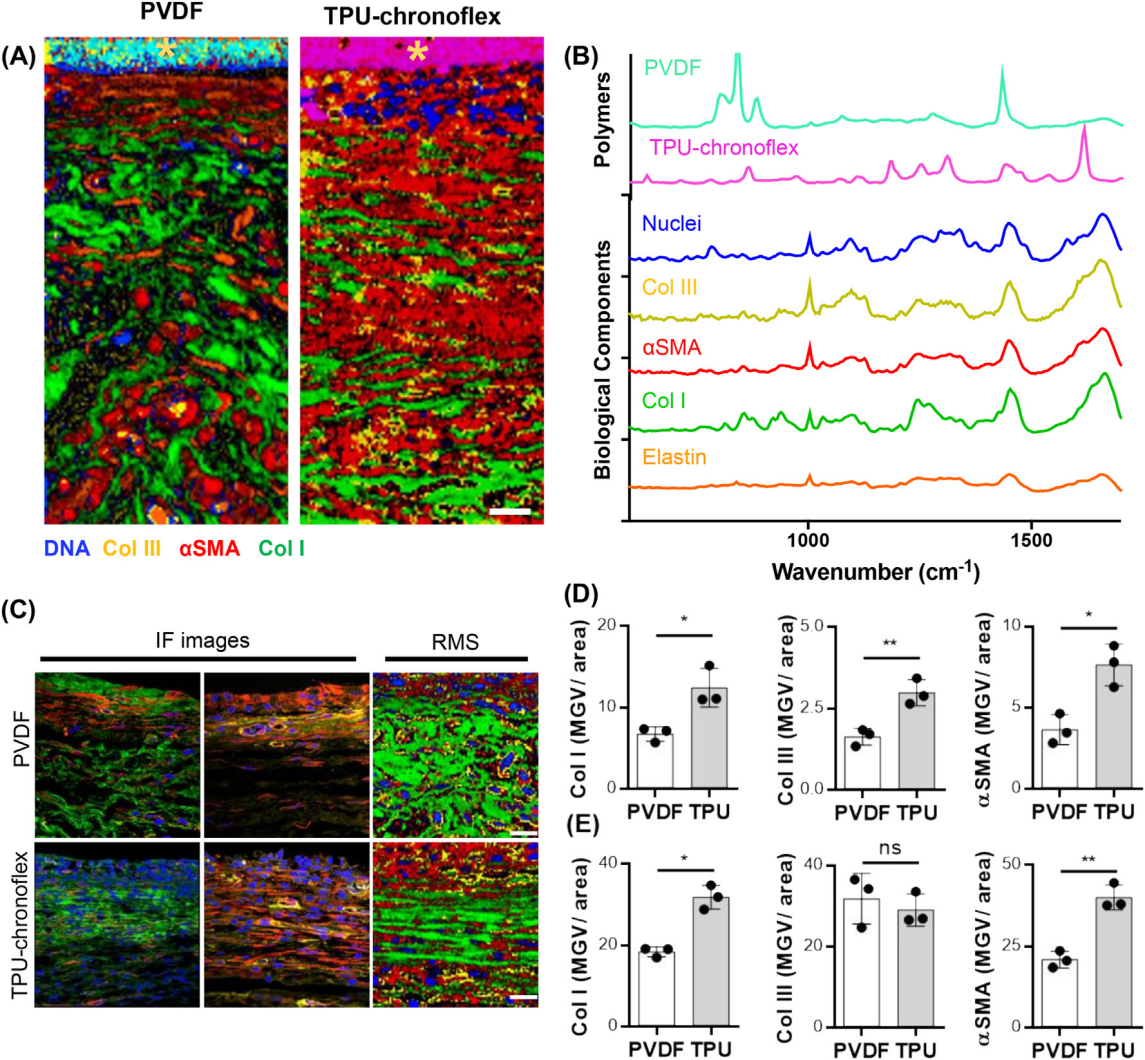
**Comparison of marker-dependent and independent methods for ECM characterization. (A)** True component analysis (TCA) was utilized to generate intensity distribution heatmaps of the fibrotic capsule area. Scale bar equals 20 ​μm. **(B)** Corresponding spectra of specific components were assigned to biomaterial (light blue & pink), nuclei (blue), Col III (yellow), αSMA (red), Col I (green) and elastin (orange). **(C)** Similar trends were obtained between immunofluorescence (IF) and Raman (RMS) images of Col I (green), αSMA (red). Scale bar equals 20 ​μm. Implant is localized at the upper border of each image. **(D)** Quantification of ECM components and αSMA in the fibrotic capsule showed similar outputs between IF and **(E)** RMS. N ​= ​3, *t*-test. (For interpretation of the references to colour in this figure legend, the reader is referred to the Web version of this article.)

Expression and deposition of fibrosis-relevant ECM components surrounding the implant were further compared between tissue sections of PVDF and TPU-chronoflex implantation via conventional and marker-independent imaging methods. Raman scans were acquired at the interface between implant and interstitial connective tissue. TCA was first applied on the Raman scans to generate color-coded intensity distribution heat maps of the most relevant structures. (Fig. 2A). According to specific spectral signatures (Fig. 2B), PVDF and TPU-chronoflex, demonstrated distinctive Raman spectra compared to other biomolecules. Col I, Col III and elastin were localized based on reference spectra obtained in a previous study [33]. The dense fibrillar collagens in the capsular area showed a parallel alignment to the implants, whereas the interstitial connective tissue region contained loosely arranged collagen fibers (Fig. 2C). These morphological observations showed correlation with SHG images (Supplementary Fig. S3). Additionally, Col I and αSMA were identified in the Raman images. Distribution of Col I and αSMA signals was more prominent in the fibrotic capsule regions compared to the interstitial connective tissue (Fig. 2C). IF staining was applied to identify Col I (green), Col III (yellow), αSMA (red), and nuclei (blue) in all tissues to validate corresponding Raman signals. (Fig. 2C). Raman scans were applied on consecutive sections without IF staining. Analogous distribution patterns of Col I and αSMA were shown in both imaging methods and confirmed the observations of histological analyses. Raman imaging was compared to IF imaging in regards of semi-quantitative analysis (Fig. 2D and E). The marker-dependent and marker independent readouts showed similar quantitative results in Col I and αSMA expression but no in Col III. In addition, no significant differences were found when comparing the ratio of Col I to Col III areas in both methods (Supplementary Fig. S3).

### 4.3 Discrimination of fibrotic capsule tissues and interstitial connective tissues via multivariate analysis of col I spectra

**Fig. 3:**
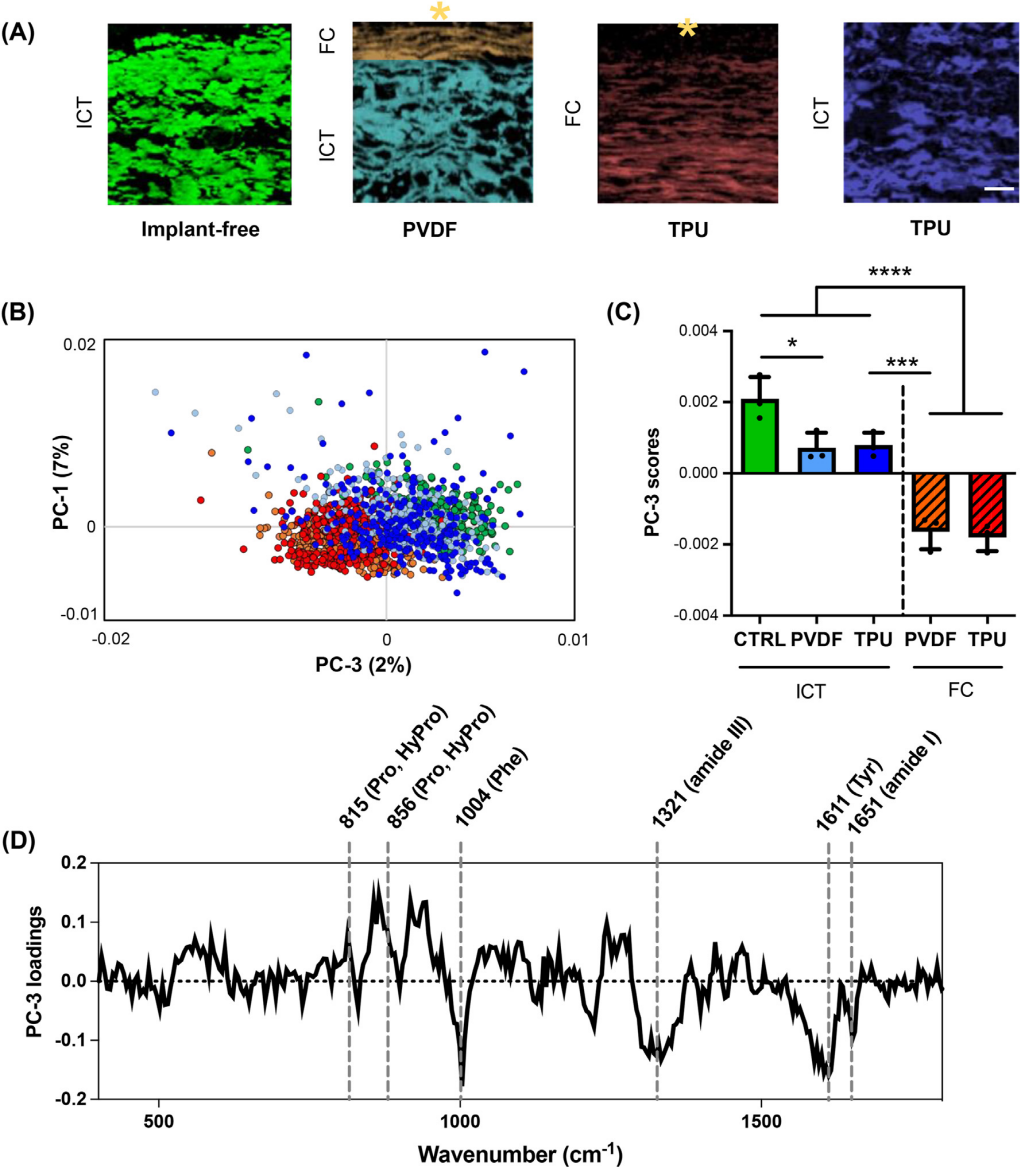
**Multivariate data analysis of Col I spectra differentiates molecular features between the fibrotic capsule and connective tissue. (A)** Col I signals were extracted from various tissue types with or without implantation. ICT: Interstitial connective tissue; FC: fibrotic capsule scale bar equals 20 ​μm. **(B)** PCA scatter plot shows a separation of Col I features between fibrotic capsule and connective. **(C)** PC-3 loading scores of Col I Raman spectra can be used to differentiate fibrotic tissues and connective tissues. N ​= ​3 per group, one-way ANOVA, p value ​< ​0.0001. **(D)** Biological assignments that contributed to spectral differences are indicated in the PC-3 loadings plot. (Hy)Pro: (Hydroxy)Proline, Phe: Phenyl alanine, Tyr: Tyrosine.

Col I morphology from the tissue surrounding the two implant types demonstrated non-identical patterns in fibrotic areas and interstitial connective tissues. In addition to marker-independent structural assignment and visualization, RMS provides molecular information of biological components reflected in spectral variations. Therefore, single Col I spectra were extracted from fibrotic and interstitial connective tissue regions to perform a PCA. Herein, the interstitial connective tissue from implant-free tissue regions of the same animals were used as a control. The PC-1/PC-3 scores plot showed two main clusters separating Col I signatures derived from interstitial connective tissues (implant-free regions and connective tissue layer below PVDF and TPU-chronoflex implants) and fibrotic capsule (the fibrotic interface adjacent to the implants) (Fig. 3A and B). PC-3 scores demonstrated significant differences between Col I extracted from the two areas. Col I spectra from interstitial connective tissues clustered at positive score ranges, whereas negative values were demonstrated for Col I from fibrotic areas (Fig. 3C). The corresponding loadings plot showed biological assignments to peaks at 1651 ​cm^−1^, 1611 ​cm^−1^, 1321 ​cm^−1^, 1004 ​cm^−1^, 856 ​cm^−1^ and 815 ​cm^−1^ (Fig. 3D), assigned to amide I, tyrosine, amide III, phenylalanine, proline and hydroxyproline. A detailed overview of the most relevant peaks and their molecular assignment is provided in Supplementary Table S1. A PCA on Col I was performed for in-depth comparison only within the fibrotic capsules and enabled to further elaborate alterations of Col I composition between PVDF and TPU-chronoflex indicated by spectral changes at 923, 972 and 1448 ​cm^−1^ (Supplementary Fig. S5).

### 4.4 Identification of M1-and M2-like macrophages via nuclei spectra

**Fig. 4:**
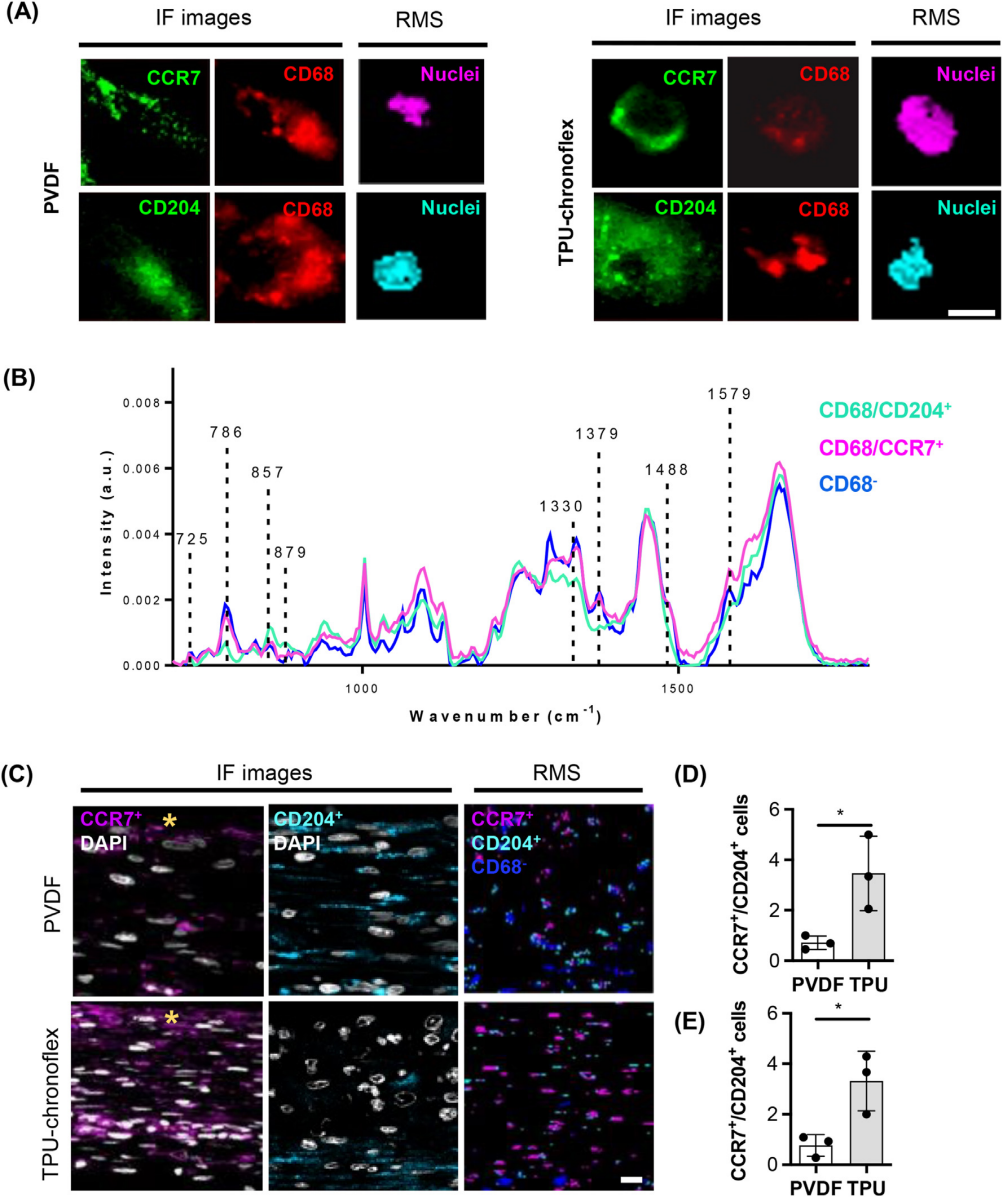
**Macrophage phenotyping via fluorescence-guided generation of reference spectra. (A)** IF staining of CD68^+^/CCR7^+^ and CD68^+^/CD204^+^ cells in PVDF and TPU-chronoflex samples was applied to target nuclei of macrophages for correlative Raman imaging (pink & light blue). Scale bar equals 3 ​μm. **(B)** Average nuclei spectra extracted from CCR7^+^ (pink) and CD204^+^ (light blue) macrophages, compared to non-macrophage nuclei spectrum (blue). **(C)** Raman spectra were applied to the previously acquired unstained Raman scans of the fibrotic capsule for a guided TCA on cellular subtypes and compared to IF images. Scale bar equals 20 ​μm; yellow asterisk indicates interface to implant. The ratio of CCR7^+^ to CD204^+^ cell numbers was significantly different between the two implantation groups for both IF **(D)** and Raman **(E)** images. N ​= ​3, *t*-test, p∗ <0.05. (For interpretation of the references to colour in this figure legend, the reader is referred to the Web version of this article.)

Polarization of tissue infiltrating macrophages towards a M1 (pro-inflammatory) or M2 (anti-inflammatory) phenotype can indirectly influence fibrosis severity [34]. IF stains were applied on tissue sections to identify different phenotypes of macrophages via fluorescence-guided acquisition of Raman reference spectra. In addition to pan-macrophage marker CD68, tissues were stained for either CCR7 or CD204 to acquire Raman scans of single cells by fluorescence guidance (Fig. 4A). The spectra co-localized with positive surface protein signals were extracted and compared to the spectrum of DNA signals from CCR7^-^/CD204^-^ cells within the fibrotic tissue. Similar signatures with subtle variations in the peaks at 1579 ​cm^−1^, 1488 ​cm^−1^, 1379 ​cm^−1^, 1330 ​cm^−1^, 879 ​cm^−1^, 857 ​cm^−1^ and 725 ​cm^−1^ could be observed among the three spectra (Fig. 4B). The identified spectra were applied as reference spectra to unstained Raman scans of PVDF and TPU-chronoflex induced fibrotic capsules and enabled the identification and localization of cellular subtypes, which showed high correlation with IF images (Fig. 4C). Based on the Raman and IF images, ratios of CCR7^+^ to CD204^+^ macrophages were determined. Differences in macrophage polarization were observed in fibrotic capsule tissue formed around PVDF and TPU-chronoflex. The ratio between CCR7^+^ to CD204^+^ cell numbers was significantly increased in FBR induced by TPU-chronoflex implantation compared to PVDF (Fig. 4D and E). Quantification based on Raman imaging was performed at similar specificity as conventional IF imaging.

### 4.5 Molecular-sensitive macrophage phenotyping via PCA analysis on nuclei spectra

**Fig. 5:**
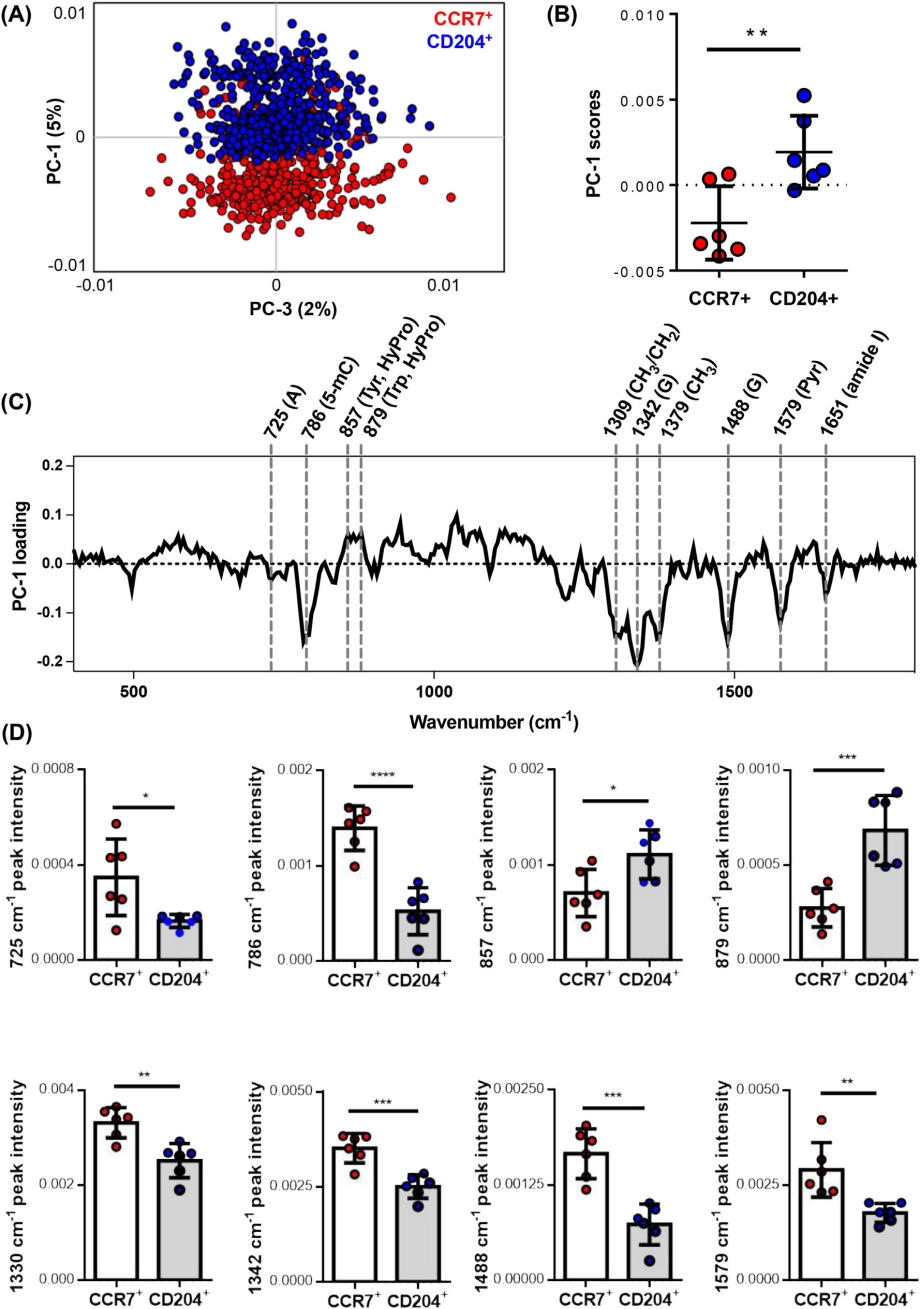
**Spectral peak analysis on nuclei signatures of M1-and M2-like macrophages. (A)** PCA comparison demonstrates distinct separation in nuclei signals between CCR7^+^ and CD204^+^ macrophages. **(B)** Average PC-1 score values of nuclei spectra differed between CCR7^+^ and CD204^+^ cells. **(C)** Biological assignments were shown in PC-1 loading plots. A: Adenine, 5-mC: 5-methylcytosine; Pyr: Tyrosine; HyPro: Hydroxyproline; Trp: Tryptophane; G: Guanine; Pyr: Pyrimidine **(D)** Statistical analysis of relative peak intensities in Raman peaks derived from CCR7^+^ and CD204^+^ cells. N ​= ​6, *t*-test, p∗< 0.05, p∗∗< 0.01, p∗∗∗< 0.001, p∗∗∗∗<0.0001.

To further investigate the biological relevance of the identified alterations in nuclei signatures of M1-like (CCR7^+^) and M2-like (CD204^+^) macrophages, spectral data from the assigned nuclei were extracted and applied for PCA to further elaborate spectral variations among the macrophage phenotypes. Two separated clusters were displayed in the PC-1 vs PC-3 scores plot (Fig. 5A). The CCR7^+^ cell derived data clustered at negative PC-1 score ranges, whereas CD204^+^ cell derived spectral data shifted towards positive PC-1 score values. PC-1 scores showed significant difference between nuclei spectra of M1-like and M2-like macrophages (Fig. 5B). In PC-1 loadings, difference in nuclei features were identified at increased Raman shifts for CCR7^+^ cells at 1651 ​cm^−1^, 1579 ​cm^−1^, 1488 ​cm^−1^, 1379 ​cm^−1^, 1342 ​cm^−1^, 1309 ​cm^−1^, 786 ​cm^−1^ and 725 ​cm^−1^ (Fig. 5C), assigned to lipids, pyrimidine, guanine, CH_3_ and CH_3_/CH_2_ twisting, 5-Methylcytosine and adenine (Supplementary Table S1). Nuclei spectra of CD204^+^ macrophages showed higher intensities at the two peaks at 879 ​cm^−1^ and 857 ​cm^−1^, corresponding hydroxyproline, proline, tryptophan and tyrosine. Statistical analysis of the most relevant loadings-derived Raman bands demonstrated significant differences in respective peak intensities of CCR7^+^ and CD204^+^ macrophage subsets (Fig. 5D).

## 5 Discussion

RMS was evaluated as a novel technique to obtain robust and faster discrimination between interstitial connective and fibrotic tissues when compared to morphological assessment of histological stains. PCA was conducted on Col I spectra and identified different biomolecular signatures for different tissue regions. Significant differences between the fibrotic and non-fibrotic tissue areas indicated secondary conformational difference in Col I. Although investigations via RMS regarding collagen conformational changes have been described in various pathological and disease states [35,36], Col I alterations between fibrotic and interstitial connective tissues have seldom been reported. In this study, Col I in the fibrotic capsule demonstrated higher spectral contributions of proline, hydroxyproline, tyrosine, phenylalanine and amide I, which might affect physical stability in its triple helical structures [37,38]. Collagen production in wound healing process highly relies on proline and hydroxyproline [39,40]. This might explain the differences at 815 ​cm^−1^ and 856 ​cm^−1^ (proline and hydroxyproline) in the Col I spectra, which showed decreased intensities in the fibrotic tissues compared to interstitial connective tissues. A recent study utilized a murine model to evaluate wound healing activity by administration of proline and hydroxyproline [41]. The results showed that proline and hydroxyproline might induce muscle regeneration with less fibrotic tissue. Though the mechanism requires more studies to be further elaborated, these differences are of great promise as potential biomarkers to identify fibrotic alterations in collagens as a proxy for immune response to implants. This finding can be expanded to further diagnostic applications. The definition of the thickness or the area of the fibrotic tissue in histological stains might cause bias between different individuals. Notably, our study highlights that fibrotic and interstitial connective tissues can be differentiated based on Raman spectrum of Col I, allowing non-biased/automated interpretation of the fibrotic regions.

Macrophages, are one of the various immune cells involved in innate and adaptive immunity [42], and play a key role in the process of FBR [43]. The interaction of macrophages and the surface of biomaterials at the initial stage of implantation has been extensively described and is highly relevant to define the biocompatibility of novel implant materials [17,43,44]. Currently, the classification of tissue-resident macrophages mainly relies on immunohistochemistry, chromogenic methods, RNA expression or flow cytometry [26,45]. To overcome limitations of these methods in regard to temporal and spatial resolution, our group recently established Raman measurements on an in vitro polarization model for monocyte-derived macrophages (MDM). Four different macrophage subsets (M0, M1 and M2a and M2c) were identified through their lipidome profiles assessed via RMS [26]. Whereas single-cell measurements in suspension provide less background noise, identification of cells in a complex tissue environment remains challenging. Not only due to the sample heterogeneity, but also due to impacts of sample processing (fixation, removal of lipids during deparaffinization) and challenging immunofluorescence-based validation of immune subsets. Multiplexing is feasible in flow cytometry based immune phenotyping of macrophage subsets but limited in conventional IF staining.

In this study, we proposed a potential approach for in situ immunophenotyping by marker-independent Raman imaging, based on fluorescence-guided generation and validation of macrophage subset reference spectra. There is a multitude of rather M1- (CD86, CCR7, CX3CR1, IL-7R, CD80, IL-1β, IL-12 and HLA-DR) or M2-like (CD163, CD204, CD206, CD209, CD369) surface markers [45,46]. In accordance to a previous study on rat tissue [[47], [48], [49]], CCR7 and CD204 were selected as IF markers for pro-inflammatory M1-like and anti-inflammatory M2-like) phenotypes to identify the regions of interest and acquire spectra of colocalized nuclei. These reference spectra allowed for the identification and discrimination of M1-like and M2-like phenotypes on non-processed subcutaneous tissue sections. The analyses demonstrated higher M1-like/M2-like cell number ratios for FBR induced by TPU-chronoflex than for PVDF. Lower M1-like/M2-like cell number ratio has been widely shown to indicate less severe inflammatory and fibrotic reaction to implants [18,50]. Moreover, M1-like macrophages showed higher intensities in Raman bands corresponding to the assignments of guanine, adenine, CH_3_/CH_2_ twisting and 5-methylcytosine (5-mC). Those peak shifts could indicate relatively higher DNA methylation state in the cells, which is consistent to our previous findings [51]. Our previous work hypothesized that Raman bands at 1257 ​cm^−1^, 1331 ​cm^−1^, 1342 ​cm^−1^ and 1579 ​cm^−1^ might be distinctive markers for DNA methylation. The M1-like vs M2-like loadings in this study demonstrated similar tendencies in the Raman bands corresponding to identical biological assignments, where significant differences in peak intensity were found at 1330 ​cm^−1^ (Twisting/wagging modes of CH_3_ and CH_3_CH_2_ in nucleic acids), 1342 (Guanine), 1488 (Guanine) and 1579 ​cm^−1^ (Pyrimidine ring). The significantly increased signal at 786 ​cm^−1^ has been reported to represent 5-mC [51,52], which is the methylated cytosine in the DNA backbone that is mainly involved in gene transcription regulation. In fact, it has been shown that epigenetic modifications, especially DNA methylation play a role in macrophage polarization. Inhibition of DNA methyltransferase 1 (DNMT1) can promote activation of M2 macrophages, resulting in anti-inflammatory function [53,54]. The increased intensities in the peaks at 857 ​cm^−1^ and 879 ​cm^−1^ in the nuclei signature of M2 macrophages were assigned to proline, hydroxyproline, tryptophan and tyrosine (Supplementary Table S1). Proline, as one of the epigenetic modifiers, is the precursor of α-ketoglutarate [55,56]. α-Ketoglutarate is capable of entering the nucleus [57], which can be utilized as a substrate of ten-eleven translocation (TET) proteins and Jumonji C domain demethylases for DNA demethylation and histone demethylation, respectively [55,58]. The high-level of proline signals in M2-like macrophages compared to M1-like macrophages explained that the M2 phenotypes had lower methylation levels than M1 macrophages. This biological phenomenon has also been shown by other groups that α-ketoglutarate can induce anti-inflammatory response by promoting macrophage polarization into M2 macrophages and suppressing M1 macrophages activation [59,60]. Apart from using the difference in the degree of DNA methylation to discriminate M1-like/M2-like phenotypes, the peak intensity at 725 ​cm^−1^ has been noted to decrease in M2-like macrophages [61]. These results were consistent to the histological and Raman images, demonstrating that compared to PVDF, tissues with TPU-chronoflex implantation induced more severe FBR.

While PVDF has been widely used in the clinic as surgical meshes [62], TPUs have been applied to cardio- or vascular implants or catheters [63]. It has been reported that the inflammatory and fibrotic effects of PVDF are less severe compared to other materials [64]. This attenuated immune response might be due to the high similarity of the carbon backbone in the molecular structure of PVDF to human tissues [62,65]. TPU also has been reported to induce relatively mild FBR [66]. Nonetheless, in our study, a modified TPU-chronoflex dual-layer pouch was applied as a candidate delivery system for islet encapsulation, which has not been characterized before. The overview of the brightfield images of Movat pentachrome staining showed the fibrotic tissue caused by TPU-chronoflex was significantly thicker than by the PVDF pouch. Picrosirius red staining has been reported for the use of examination of the scar collagen states such as fiber thickness or maturation [30,67,68]. Picrosirius red staining demonstrated difference in the organization of collagen fibers between the fibrotic capsule and interstitial connective tissues. Nevertheless, the definite indication of the colorimetry in PSR staining with polarized light remains controversial and requires complementary validations. The morphological characterization of the fibrotic capsules was followed by IF staining and Raman imaging of implant-adjacent tissue regions. ECM remodeling in FBR involves immune cell recruitment, cytokine and chemokine release as well as collagen deposition [69]. Previous work by our group has shown the great potential of RMS to investigate ECM structures and biomolecules in tissue engineering [23,70,71]. Based on established reference signatures, αSMA, Col I and Col III were identified and localized in the fibrotic capsule. Quantitative evaluation of Raman imaging demonstrated the TPU-chronoflex group had significantly higher Col I production level. The latter value might indicate an increased Col I secretion by myofibroblasts in the TPU-chronoflex group. Furthermore, the enhanced level of Col I in TPU-chronoflex group could indicate an advanced stage of fibrillogenesis compared to the PVDF group [72].

This proof-of-principle study demonstrated a potential way to evaluate the foreign body response by using RMS in combination with large database and machine learning. Current approaches for fibrotic tissues assessment in preclinical research as well as clinical diagnosis still highly rely on conventional histopathological analysis and IF staining for ECM characterization [[73], [74], [75]]. These examinations demand invasive tissue biopsy procedures and represent endpoint readouts. MRI, CT, SHG and ultrasound elastography provide optical examination on architectural characteristics of fibrosis [76,77]; however, these investigations are confined to morphological differences, geometrical and quantitative evaluation of collagen fibers, that mainly indicate late-stage fibrotic tissues. These advanced techniques are inadequate to monitor the progression of the fibrotic tissue formation in a molecular manner [78]. Here, RMS can be installed on an intraoperative scope to assist clinical evaluation of pathologies [79,80]. Our results and the identified spectral biomarkers for fibrotic alterations and cellular polarization encourage follow-up studies to implement label-free, non-invasive and real-time monitoring of capsular progression after implantation.

## 6 Conclusion

Raman imaging and microspectroscopy were shown to specifically identify and characterize FBR towards two biomaterial pouches, validated by conventional histological standards. Herein, we showed that RMS combined with multivariate analysis can provide marker-independent and molecular-sensitive methods to characterize and visualize ECM proteins in tissue samples and distinguish a fibrotic capsule from normal tissue according to compositional differences in Col I spectra. This is the first study applying RMS for M1 and M2 macrophage phenotyping in ex vivo tissues and holds great potential for the future use of clinical, non-destructive investigation of FBR.

## Credit author statement

C.-E.L.: Conceptualization, Writing – original draft, Methodology, Validation, Formal analysis, Investigation, Visualization. R.E.L.: Methodology. G.G.: Methodology. N.S.: Investigation, Methodology. S.L.: Methodology. S.L.L.: Writing – review & editing. K. S.-L.: Conceptualization, Funding acquisition, Supervision, Project administration. G.P.D.: Conceptualization, Funding acquisition, Supervision, Project administration. J.M.: Conceptualization, Methodology, Supervision, Writing – review & editing.

## Declaration of competing interest

The authors declare the following financial interests/personal relationships which may be considered as potential competing interests: Katja Schenke-Layland reports financial support was provided by German Research Foundation. Katja Schenke-Layland & Garry Duffy reports financial support was provided by 10.13039/501100000780European Union. Chuan-En Lu reports equipment, drugs, or supplies was provided by Boston Scientific Ireland Ltd. Katja Schenke-Layland reports financial support was provided by State of Baden-Wurttemberg Ministry for Science Research and Art. Giulio Ghersi reports a relationship with Abiel Sr that includes: employment.

## Data Availability

Data will be made available on request.
