# Supplementary material for: Monitoring the macrophage response towards biomaterial implants using label-free imaging
Source: Mater Today Bio. 2023 Jun 13;21:100696. doi: 10.1016/j.mtbio.2023.100696 (PMC10285553; doi:10.1016/j.mtbio.2023.100696)
Supplement: Multimedia component 1 [file mmc1.pdf]

Supplementary Information

Monitoring the Macrophage Response to Biomaterial Implants Ex vivo Using Raman Microspectroscopy

Chuan-en Lu[1], Ruth E Levey[2], Giulio Ghersi[3,4], Nathan Schueller[1], Simone Liebscher[1], Shannon L Layland[1], Katja Schenke-Layland[1, 5, 6], Garry P Duffy[2, 7], Julia Marzi[1, 5, 6*]

Raman shifts (cm^{-1})	Biological assignment
1651	Amide I[1]
1623	Tryptophan[2]
1611	Tyrosine[3]
1579	Pyrimidine ring[4]
1488	Guanine (N7)[5]
1448	Collagen[6]
1379	CH_3[4]
1342	Guanine[7]
1330	DNA and phospholipids[8, 9]
1321	Amide III (α-helix)[10]
1309	CH_3/CH_2 twisting[11]
1114	NH_3[12]
1043	Proline[13]
1004	Phenylalanine[11]
972	C-C backbone[13]
923	C-C backbone[13]
879	Tryptophan, Hydroxyproline[11]
857	Tyrosine, hydroxyproline[11, 14]
856	Proline, hydroxyproline[11]
815	Proline, hydroxyproline[11]
786	5-Methylcytosine[15, 16]
725	Adenine[17]

Supplementary Table 1: Biological assignments of relevant Raman peaks.

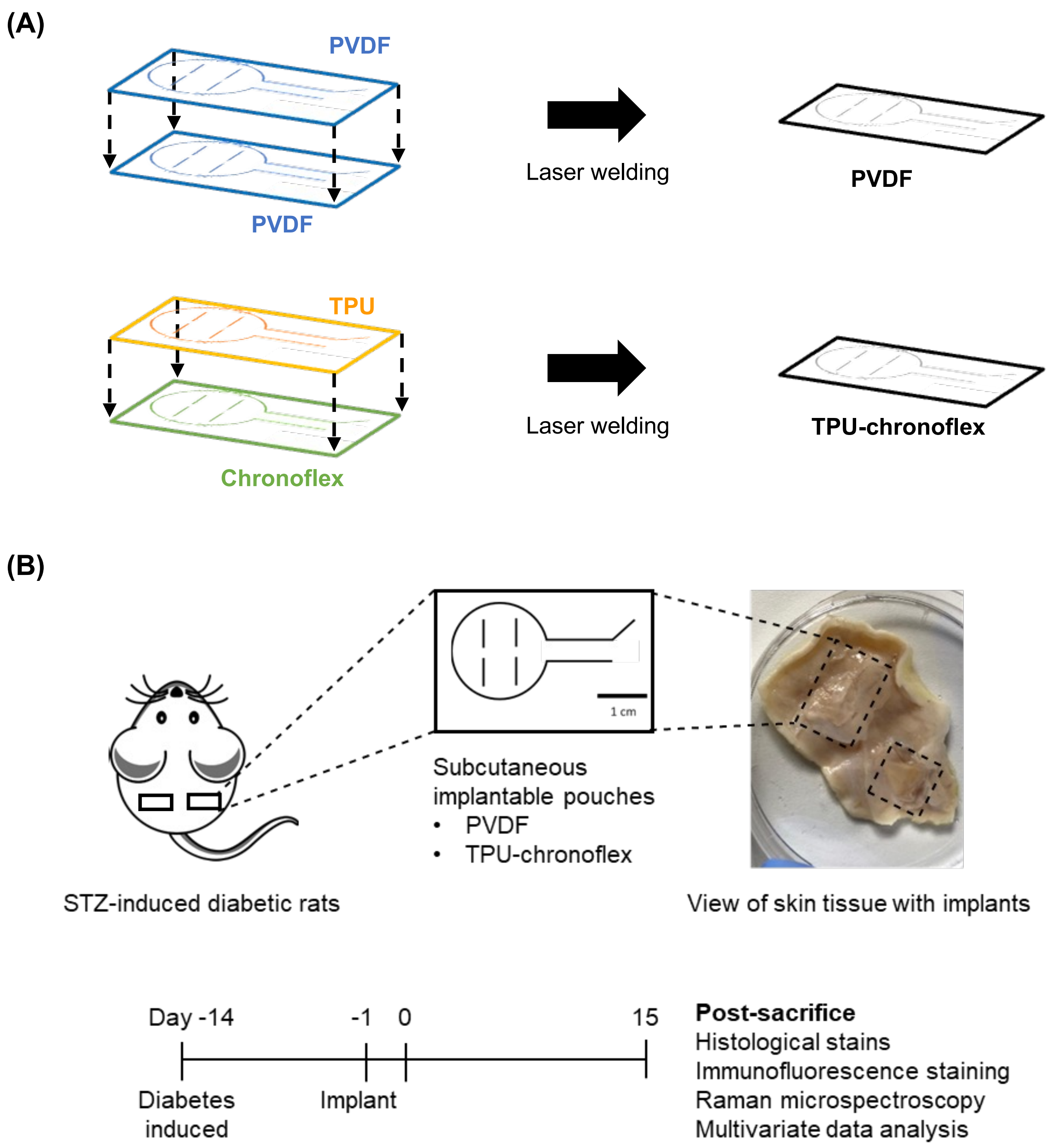

Supplementary figure 1. Implantable pouches and scheme of study design. (A) Fabricated sheets, PVDF and TPU-chronoflex with 50 µm thickness and 2 µm pore size, generated by laser-welding method. **(B)** Diabetes was induced by utilizing STZ 14 days before implantation. PVDF and TPU-chronoflex pouches were implanted subcutaneously at the back of the diabetic rats over 15 days.

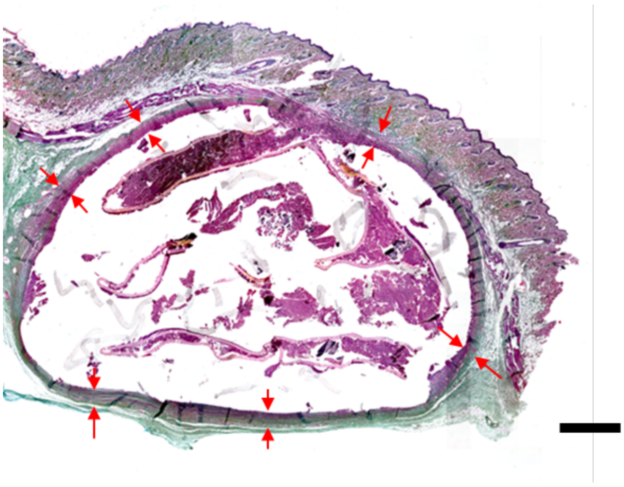

Supplementary figure 2. Depiction of capsular thickness measurement from three regions of fibrotic capsule at the upper layer of the implant and 3 sections from the bottom. scale bar equals 1 mm.

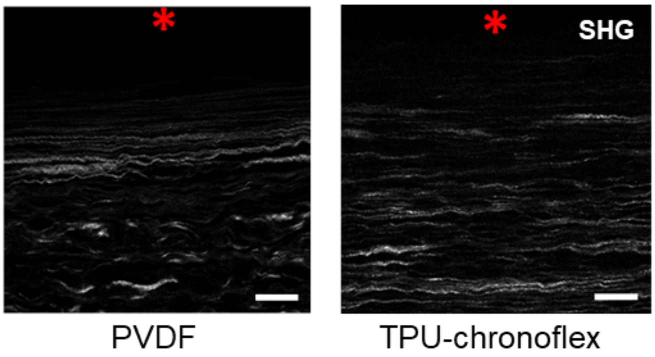

Supplementary figure 3. Second harmonic generation (SHG) signals of Collagens in PVDF and TPU-chronoflex groups. Red asterisk: implant side. Scale bars = 20 µm.

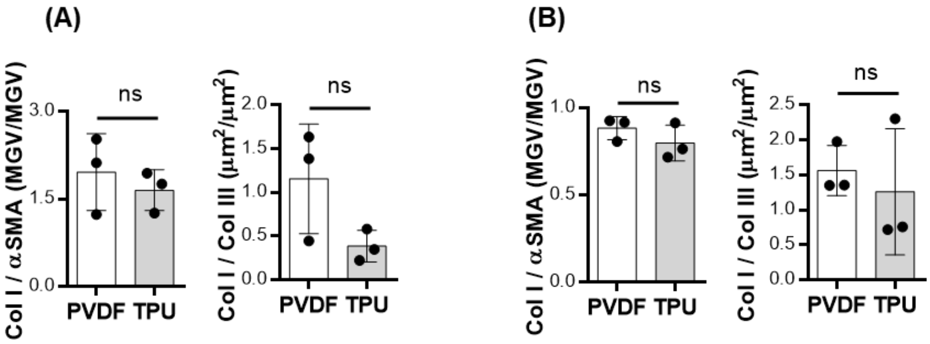

Supplementary figure 4. Ratio Quantification of Col I, Col III and αSMA in the fibrotic capsule. (A) IF imaging (B) Raman imaging. N=3, t test, $p^* \leq 0.05$.

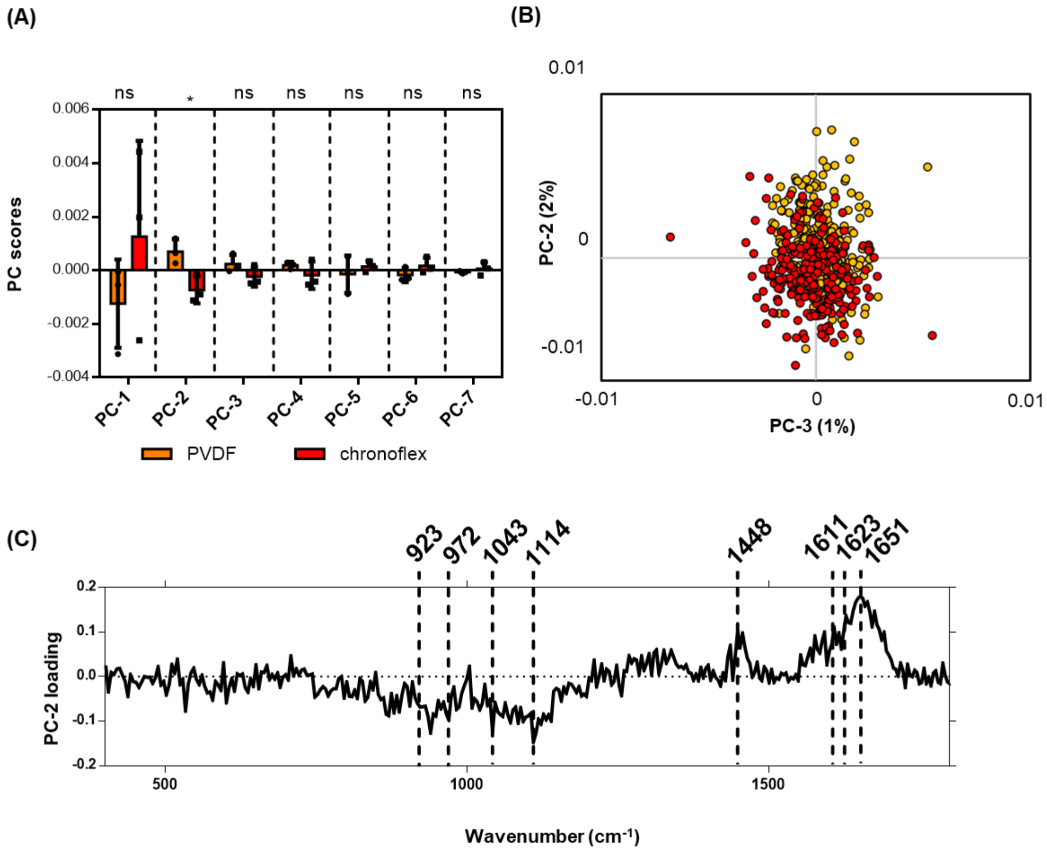

Supplementary Figure 5. Multivariate analysis discriminates Col I composition in PVDF and TPU-chronoflex samples. (A) Average PC-score values of Col I spectra indicate significant difference of fibrotic tissues between two implants in PC-2. n=3, p*< 0.05. **(B)** PC-2 vs PC-3 scores plot shows a separation in fibrotic capsule Col I features between PVDF and TPU-chronoflex. **(C)** PC-2 loadings identifies relevant spectral changes in Col I spectra.

References

[1] P.J. Caspers, H.A. Bruining, G.J. Puppels, G.W. Lucassen, E.A. Carter, In Vivo Confocal Raman Microspectroscopy of the Skin: Noninvasive Determination of Molecular Concentration Profiles, Journal of Investigative Dermatology 116(3) (2001) 434-442.
[2] N. Huang, M. Short, J. Zhao, H. Wang, H. Lui, M. Korbelik, H. Zeng, Full range characterization of the Raman spectra of organs in a murine model, Opt Express 19(23) (2011) 22892-909.
[3] H. Abramczyk, A. Imiela, B. Brożek-Płuska, M. Kopeć, J. Surmacki, A. Śliwińska, Aberrant Protein Phosphorylation in Cancer by Using Raman Biomarkers, Cancers 11(12) (2019) 2017.
[4] N. Stone, C. Kendall, J. Smith, P. Crow, H. Barr, Raman spectroscopy for identification of epithelial cancers, Faraday Discuss 126 (2004) 141-57; discussion 169-83.
[5] A. Ruiz-Chica, M. Medina, F. Sánchez-Jiménez, F. Ramírez, Characterization by Raman spectroscopy of conformational changes on guanine–cytosine and adenine–thymine oligonucleotides induced by aminooxy analogues of spermidine, Journal of Raman Spectroscopy 35 (2004) 93-100.
[6] S. Kaminaka, H. Yamazaki, T. Ito, E. Kohda, H.-o. Hamaguchi, Near-infrared Raman spectroscopy of human lung tissues: possibility of molecular-level cancer diagnosis, Journal of Raman Spectroscopy 32(2) (2001) 139-141.
[7] Z. Liu, C. Davis, W. Cai, L. He, X. Chen, H. Dai, Circulation and long-term fate of functionalized, biocompatible single-walled carbon nanotubes in mice probed by Raman spectroscopy, Proceedings of the National Academy of Sciences 105(5) (2008) 1410-1415.

[8] U. Utzinger, D.L. Heintzelman, A. Mahadevan-Jansen, A. Malpica, M. Follen, R. Richards-Kortum, Near-Infrared Raman Spectroscopy for in Vivo Detection of Cervical Precancers, Appl. Spectrosc. 55(8) (2001) 955-959.
[9] R. Malini, K. Venkatakrishna, J. Kurien, K.M. Pai, L. Rao, V.B. Kartha, C.M. Krishna, Discrimination of normal, inflammatory, premalignant, and malignant oral tissue: a Raman spectroscopy study, Biopolymers 81(3) (2006) 179-93.
[10] R. Jyothi Lakshmi, V.B. Kartha, C. Murali Krishna, R.S. JG, G. Ullas, P. Uma Devi, Tissue Raman spectroscopy for the study of radiation damage: brain irradiation of mice, Radiat Res 157(2) (2002) 175-82.
[11] W.T. Cheng, M.T. Liu, H.N. Liu, S.Y. Lin, Micro-Raman spectroscopy used to identify and grade human skin pilomatrixoma, Microsc Res Tech 68(2) (2005) 75-9.
[12] K.I. Hadjiivanov, D.A. Panayotov, M.Y. Mihaylov, E.Z. Ivanova, K.K. Chakarova, S.M. Andonova, N.L. Drenchev, Power of Infrared and Raman Spectroscopies to Characterize Metal-Organic Frameworks and Investigate Their Interaction with Guest Molecules, Chemical Reviews 121(3) (2021) 1286-1424.
[13] F. Bonnier, H.J. Byrne, Understanding the molecular information contained in principal component analysis of vibrational spectra of biological systems, Analyst 137(2) (2012) 322-32.
[14] C.A. Lieber, H.E. Nethercott, M.H. Kabeer, Cancer field effects in normal tissues revealed by Raman spectroscopy, Biomed. Opt. Express 1(3) (2010) 975-982.
[15] R. Daum, E.M. Brauchle, D.A.C. Berrio, T.P. Jurkowski, K. Schenke-Layland, Non-invasive detection of DNA methylation states in carcinoma and pluripotent stem cells using Raman microspectroscopy and imaging, Scientific Reports 9(1) (2019) 7014.
[16] A. Barhoumi, N.J. Halas, Detecting Chemically Modified DNA Bases Using Surface-Enhanced Raman Spectroscopy, The Journal of Physical Chemistry Letters 2(24) (2011) 3118-3123.
[17] J.W. Chan, D.S. Taylor, T. Zwerdling, S.M. Lane, K. Ihara, T. Huser, Micro-Raman spectroscopy detects individual neoplastic and normal hematopoietic cells, Biophys J 90(2) (2006) 648-56.
